# Polyelectrolyte Based Sensors as Key to Achieve Quantitative Electronic Tongues: Detection of Triclosan on Aqueous Environmental Matrices

**DOI:** 10.3390/nano10040640

**Published:** 2020-03-29

**Authors:** Cátia Magro, Paulo Zagalo, João Pereira-da-Silva, Eduardo Pires Mateus, Alexandra Branco Ribeiro, Paulo Ribeiro, Maria Raposo

**Affiliations:** 1 CENSE, Departamento de Ciências e Engenharia do Ambiente, Faculdade de Ciências e Tecnologia, Universidade Nova de Lisboa, 2829-516 Caparica, Portugal; epm@fct.unl.pt (E.P.M.); abr@fct.unl.pt (A.B.R.); 2 CEFITEC, Departamento de Física, Faculdade de Ciências e Tecnologia, Universidade Nova de Lisboa, 2829-516 Caparica, Portugal; zagalojunior@hotmail.com (P.Z.); joaosilva473@gmail.com (J.P.-d.-S.); pfr@fct.unl.pt (P.R.)

**Keywords:** environmental monitoring, triclosan, layer-by-layer technique, interdigitated sensors, electronic tongue, impedance spectroscopy

## Abstract

Triclosan (TCS) is a bacteriostatic used in household items that promotes antimicrobial resistance and endocrine disruption effects both to humans and biota, raising health concerns. In this sense, new devices for its continuous monitoring in complex matrices are needed. In this work, sensors, based on polyelectrolyte layer-by-layer (LbL) films prepared onto gold interdigitated electrodes (IDE), were studied. An electronic tongue array, composed of (polyethyleneimine (PEI)/polysodium 4-styrenesulfonate (PSS))_5_ and (poly(allylamine hydrochloride/graphene oxide)_5_ LbL films together with gold IDE without coating were used to detect TCS concentrations (10^−15^–10^−5^ M). Electrical impedance spectroscopy was used as means of transduction and the obtained data was analyzed by principal component analysis (PCA). The electronic tongue was tested in deionized water, mineral water and wastewater matrices showing its ability to (1) distinguish between TCS doped and non-doped solutions and (2) sort out the TCS range of concentrations. Regarding film stability, strong polyelectrolytes, as (PEI/PSS)_n_, presented more firmness and no significant desorption when immersed in wastewater. Finally, the PCA data of gold IDE and (PEI/PSS)_5_ sensors, for the mineral water and wastewater matrices, respectively, showed the ability to distinguish both matrices. A sensitivity value of 0.19 ± 0.02 per decade to TCS concentration and a resolution of 0.13 pM were found through the PCA second principal component.

## 1. Introduction

Triclosan (5-chloro-2-(2,4-dichlorophenoxy) phenol) (TCS) is widely used as an antimicrobial, antibacterial and preservative agent in different personal care and consumer products [1]. TCS’s main physical and chemical characteristics are presented in Table S1. Structurally, the TCS molecule has functional groups for both phenol (5-chloro-2-(2,4-dichlorophenoxy) phenol) and ether (2,4,4-trichloro-2-hydroxydiphenyl ether) and its lipophilicity (log K_ow_ = 4.8) resulting in a potential for bioaccumulation. Several studies had proved its allergy risk, antimicrobial resistance [2], developmental toxicity, and endocrine disruption, both in humans and biota [3,4,5,6,7,8,9]. Due to its characteristics and inefficient removal in the wastewater treatment plants, TCS and its bioactive metabolites and/or by-products, have been detected in wastewaters and superficial waters, at 10^−8^–10^−12^ M concentrations range (2 ng L^−1^–40 μg L^−1^; Table S2 Supplementary Material) [10,11,12]. Consequently, TCS is continually being introduced in the aquatic environment via a number of routes, primarily by both untreated and treated wastewater [13].

Currently, the monitoring of TCS is a matter of concern and an important issue. Traditional analytical methods use gas or liquid chromatography and mass spectrometry [14,15,16,17,18,19]. However, these methods in spite of their high reliability are rather complex, time consuming and expensive [20]. Subsequently, there is a need for rapid, low-cost, and sensitive tools for real time monitoring of TCS in environmental matrices [21,22,23,24]. Electronic tongue (e-tongue) systems present a great potential to compete, complement, or replace traditional analytical techniques. Due to their specificities: fast response times, and user-friendly operation [25,26], these devices have been used for different applications in the food industry: discrimination of the honey botanic origin [27] or in the pharmaceutical industry [28] with active pharmaceutical ingredients detection [29,30]. An e-tongue is “a multi-sensory system, formed by an array of low-selective sensors, combined with advanced mathematical procedures for signal processing, based on pattern recognition and/or multi-variate data analysis” [31]. TCS is one of the most studied pollutants in sensors development, as examples: a strand of carbon fibers with a voltammetric detector for high-performance liquid chromatography to detect TCS in rabbit serum and urine [32]; an electropolymerizing *o*-phenylenediamine on a glassy carbon electrode, using an amperometric sensor achieved a linear range of 2.0 × 10^−7^–3.0 × 10^−6^ mol/L and a detection limit of 8.0 × 10^−8^ mol/L [22]; a multiwall carbon nanotube film was developed for TCS detection using a electrochemical sensor with a linear range from 50 to 1.75 mg L^−1^, and a limit of detection of 16.5 μg L^−1^ (57 nM) [33]. Recent studies [34,35,36,37,38] have reported well succeeded applications with sensors composed of layer-by-layer (LbL) thin-films, which were produced with polyelectrolytes and used to detect organic compounds (in ultrapure water matrices and methanol) using impedance measurements.

Accordingly, the LbL nanoassembly technique will produce nanostructures, in a flexible, easily-scalable, reproducible, and versatile approach that allows the precise control of the coating thickness, composition, and structure [39]. This nanoassembly technique is a powerful tool for the incorporation of a wide variety of coating types, such as polyelectrolytes, biological molecules, liposomes, ceramics, and electrically charged small molecules [40]. An impedance system, in a simpler conceptual implementation, is the full scan of different alternated current frequencies or a selected number of discrete frequency values, which can be used (conductivity or capacitance) [41]. The impedance technique approach is based on the electrode perturbation caused by an external signal of small magnitude [27]. Measurements can be performed in the equilibrium or stationary state. There is an increased interest in the impedantiometric systems to the electrical characterization of environmental matrices, since they present simple and flexible measurements, are portable and user friendly, and a non-destructive technique [42]. Thus, the characterization of aqueous environmental matrices is performed by analyzing the electrical impedance components (reactance, resistance, capacitance, and loss tangent) as a function of frequency signals applied to nanostructures adsorbed onto solid substrates with interdigitated electrodes [37]. It has been reported that the TCS adsorbed amount per unit of area on thin-films is reduced if the outer layers have negative charged polyelectrolyte [43]. The stability of the sensorial layers produced by the LbL technique is dependent of the solution’s pH and the degree of ionization of the uppermost polyelectrolyte layer [44]. Consequently, the TCS pKa (see Table S1) will play an essential role in its adsorption or non-absorption onto the used thin-films.

**Figure 1:**
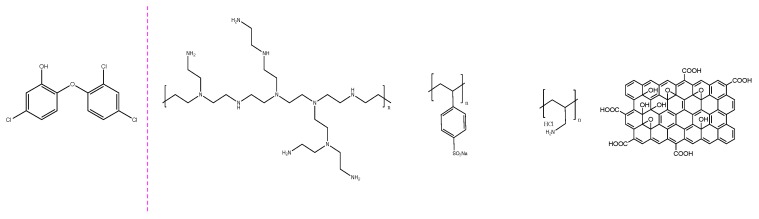
Chemical structure of triclosan and the polyelectrolytes: polyethyleneimine (PEI), poly (sodium 4-styrenesulfonate) (PSS), poly(allylamine hydrochloride) (PAH), and graphene oxide (GO).

The aim of the present study was to explore the potential of the e-tongue concept using impedantiometric detection of TCS in environmental complex aqueous matrices. A deionized water, a Portuguese mineral water, and an effluent from a wastewater treatment plant (considered the primary source of TCS into water bodies) were used as experimental matrices. TCS was spiked to the aqueous matrices and measured using thin-films sensors based on polyethyleneimine (PEI), poly(sodium 4-styrenesulfonate) (PSS), poly(allylamine hydrochloride) (PAH), and graphene oxide (GO; Figure 1), which have been prepared with the LbL technique onto solid supports with deposited gold electrodes. To the best of our knowledge, regarding wastewater real samples and these two combinations of thin-films sensors, this is the first study that explores: 

(1) an e-tongue based in a set of five sensor devices providing the possibility to distinguish different TCS concentrations;

(2) the use of electrical impedance spectroscopy measurements for TCS detection in environmental aqueous complex matrices;

(3) the stability of the thin-films layers, analysis of adsorption and desorption phenomena, submitted to different aqueous matrices and pH values.

## 2. Materials and Methods 

The experimental matrices were deionized water (DW), a Portuguese mineral water (MW), and a wastewater (EF). DW was produced using a Millipore system (Bedford, MA, USA), MW was a commercial Portuguese mineral water and EF was the liquid fraction collected in the secondary clarifier at a wastewater treatment plant (Lisbon, Portugal). Table 1 presents the characterization of the experimental matrices for conductivity, pH, and total ion concentration parameters. These features were measured because (1) the pH of aqueous solutions can lead to desorption phenomena of thin-film layers, as well as, may change the TCS degree of ionization (dissociation constant pKa = 7.9 [45]): from Table 1 one can observe that the studied matrices present pH values in the range of 6.1–8.4; and (2) ion species and respective concentration can interact with the outermost layer of the sensor’s thin-films or with the gold in the gold IDE. All these measured parameters play a crucial role in the stability of the thin-film sensors and matrix–sensor interactions with the TCS molecule (pKa 7.9). The pH was measured with a Radiometer pH-electrode EDGE (HANNA, USA). The conductivity values were measured in a Radiometer Analytic LAQUA twin (HORIBA Ltd., Japan). Ca, Cu, K, Mg, Na, P, S, and Zn were determined by inductively coupled plasma with optical emission spectrometry (ICP-OES; HORIBA Jobin-Yvon Ultima, Japan). Cl^−^ and SO_4_^2−^ were analyzed by ion chromatography (IC; DIONEX ICS-3000, USA), equipped with a conductivity detector. 

Triclosan (TCS ≥ 97%) and methanol (MeOH; gradient grade) used were from Sigma–Aldrich (Steinheim, Germany). The experimental TCS dilutions range, 10^−5^–10^−15^ M, was made sequentially from a mother solution with a concentration of 10^−4^ M and analyzed immediately after preparation. All dilutions were prepared using experimental matrices/MeOH (9:1) solutions. A solution of each experimental matrix/MeOH, without TCS, was used as the blank standard (0 M).

The sensor devices used in this work were purchased from DropSens (Llanera Asturias, Spain) and were constituted by glass BK7 solid support with deposited gold interdigitate electrodes (IDE) comprising of 250 “fingers” each (Figure S3.1 in Supplementary Data). The supports’ dimensions were 22.8 mm × 7.6 mm × 0.7 mm and each “finger” had 5 µm of width, which is the same spacing between the “fingers”. An array of sensor devices without and with different polyelectrolyte thin-films deposited onto IDE surface was used to detect the TCS in aqueous solutions. The deposited thin-films were prepared with polyethyleneimine (PEI), poly(sodium 4-styrenesulfonate) (PSS), poly(allylamine hydrochloride) (PAH), and graphene oxide (GO) polyelectrolytes, all from Sigma–Aldrich (St Louis, MO, USA), by the LbL technique [46]. Accordingly, thin-films of PAH/GO and of PEI/PSS deposited on BK7 solid support with gold IDE were obtained by adsorbing alternate layers of electrically charged polyelectrolytes at the solid/liquid interface, washing away with water the already adsorbed layers after being immersed in the polyelectrolyte solution in order to remove any polyelectrolyte molecules that were not completely adsorbed. The polyelectrolytes were made with a monomeric concentration of 10^−2^ M diluted in water Type I, produced with a Millipore system (Bedford, MA, USA). The adsorption time period of each layer (i.e., immersion time in each polyelectrolyte solution) was 30 s and, after the adsorption of each layer, the thin-film was dried using nitrogen gas. Films of PAH/GO were prepared with 5 bilayers, (PAH/GO)_5_, while films of PEI/PSS were prepared with 5, 10, and 20 bilayers, designated by (PEI/PSS)_5_, (PEI/PSS)_10_, and (PEI/PSS)_20_, respectively. Table 2 lists the thin-films used to characterize the aqueous solutions matrices under study (see Figure S3.2 on Supplementary Data: the experimental scheme that complements Table 2).

The electrical analysis of aqueous matrices was performed by measuring the impedance spectra of these sensor devices when immersed in the aqueous matrices with different TCS concentrations with a Solartron 1260 Impedance Analyzer in the frequency range of 1–1000,000 Hz, applying an AC voltage of 25 mV. To avoid contamination of the sensor devices, the impedance spectra of thin-films sensors and gold IDE were recorded for the TCS experimental matrices in a sequence of increasing concentrations from 0 to 10^−5^ M. All measurements were performed at room temperature 25 °C.

The stability of thin-films on the different experimental matrices was also studied by measuring the ultraviolet-visible (UV-vis) spectra of the LbL thin-films before and after being immersed in the experimental matrices/MeOH solutions spiked with 10^−9^ M of TCS—an average concentration found in wastewater matrices and sufficient to see changes in the UV-vis spectra. The absorbance spectra were attained using a double beam spectrophotometer UV-2101PC (Shimadzu) with a sampling interval between 800 and 200 nm, with a resolution of 0.5 nm. For each thin-film combination, three absorbance spectra were obtained: (1) before being immersed in the TCS solution (t = 0 min) to establish a baseline, (2) after five minutes of immersion (t = 5 min), and (3) after a cumulative immersion time of ten minutes (t = 10 min). Characterization of LbL thin-films stability adjusting the EF’s pH with (1:1) HNO_3_, for pH values of 3, 6, and 8, were also performed.

Principal component analysis (PCA) was carried out, regarding the normalized (Z normalization) impedance spectroscopy data, to reduce the size of data and to obtain a new space of orthogonal components, in which different concentration patterns can be observed and explained. Additionally, an array of sensors, composed by all the thin-films and gold IDE sensors, was analyzed as an e-tongue for TCS detection in EF experimental matrix. The ANOVA was performed at 95% of confidence (*p* < 0.05)*,* concerning the Table 1 data, to prove that they are statistically different.

## 3. Results and Discussion

### 3.1. Impedance Spectroscopy Measurements: Sensor Response

**Figure 2:**
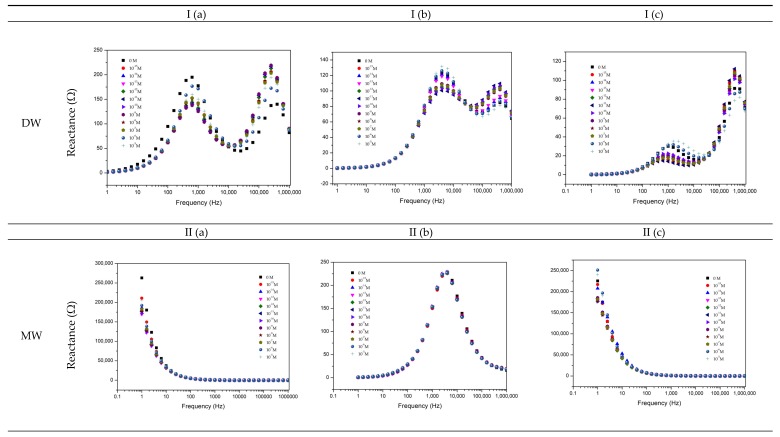

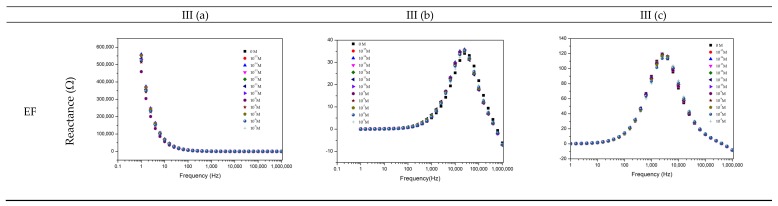
Reactance (imaginary) impedance spectra of the sensor device (**a**) of the gold IDE sensor, (**b**) of the (PAH/GO)_5_ sensor, and (**c**) of the (PEI/PSS)_5_ sensor, immersed in TCS DW (**I**), MW (**II**), and EF (**III**) at different TCS concentrations.

To evaluate the prepared sensors’ ability to detect TCS, impedance spectra were measured. As demonstrated by Taylor and Macdonald [47], the electrical properties of each thin-film, deposited on the IDE, when immersed in the aqueous sample, are dependent on the characteristics of the thin-film used as a sensitive layer, of the double layer formed on the surface of all the thin-films and of the bulk electrolyte. These components can be considered an electrical circuit. Thus, representing the equivalent circuit of the electrical characteristics of the thin-film, double-layer, and electrolyte, Figure 2 shows the imaginary impedance spectra, also designated as electrical reactance, measured by the different sensor devices when immersed in the different aqueous matrices doped with TCS concentrations from 0 to 10^−5^ M. The data points are related to the average of three impedimetric measurements (reproducibility in Supplementary Material Figure S4 for the lowest TCS concentration).

**Figure 3:**
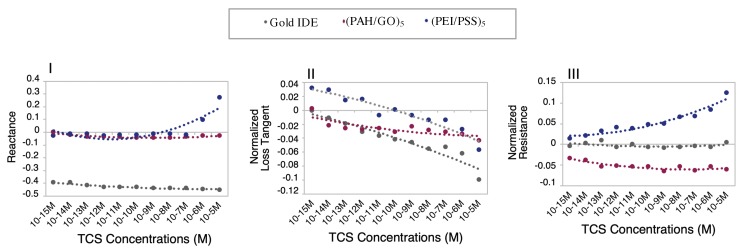
Normalized transducing variables as a function of TCS concentrations at fixed frequencies: I) DW—reactance spectra at 6.3, 25,119, and 25,119 Hz; II) MW—loss tangent spectra at 63,095, 16,000, and 100,000 Hz; and III) EF—resistance spectra at 100,000, 16,000, and 3981 Hz; to gold IDE, (PAH/GO)_5_ and (PEI/PSS)_5_ sensors, respectively (*n* = 3, average and standard deviations of the respective impedance data used for the normalization, in Supplementary Material Figures S5.1–5.3).

Different footprints were observed according to the experimental aqueous matrices and sensor devices. Similar behavior was observed for DW for all sensors. The gold IDE sensor presents a similar footprint for the MW and EF matrices. The (PAH/GO)_5_ sensor shows a similar behavior, but different from the gold IDE, for the same matrices. The (PEI/PSS)_5_ sensor shows different behavior for MW and EF matrices, however similar to the gold IDE and (PAH/GO)_5_ sensor, for MW and EF matrices, respectively. Sensor physical properties (PP), such as reactance, loss tangent, and resistance at constant frequency, may present increasing or decreasing monotone functions as a function of TCS concentration. In order to allow operational TCS measurements (sensor sensitivity), a trend function of its concentration must be found, for example at a given frequency, expressed as normalized reactance, loss tangent, or resistance spectra. A normalized response can be achieve using the following relation:

(1) $$\frac{PP\left(C\right)-PP\left(0M\right)}{PP\left(0M\right)},$$

where PP(C) is the physical property measured at a given concentration, and PP(0 M) is the physical property measured at a reference solution for the different type of matrices (TCS 0 M in DW, MW, or EF). Plots I, II, and III of Figure 3 present examples of the normalized PP spectra to fixed frequencies, for the different films and type of environmental aqueous matrices. These fixed frequency values were chosen considering which frequency represented better the curve that relates the TCS effects on which type of sensors. 

The accomplished detailed analysis of obtained spectra in TCS solutions prepared with all the experimental matrices, Figure 2, shows that the imaginary impedance or reactance could be used as a transducing variable. Regarding the DW matrix (Figure 2Ia,b,c), the gold IDE sensor did not show a clear trend, compared to the thin-films sensors that present a more pronounced sensitivity to discriminate different concentrations in the frequency ranges of 1–10 kHz, and of 100–1000 kHz. Thus, in order to better analyze the response of these sensors, the reactance values measured in the gold IDE, and (PAH/GO)_5_ and (PEI/PSS)_5_ sensors, at frequencies of 6.3 and 25,119 Hz, respectively, were normalized and plotted as a function of TCS concentrations in Figure 3I. This figure revealed that the reactance at these frequencies tended to or either showed no tendency or increase depending on the type of sensor for the TCS concentration ranges under study. The gold IDE sensor appeared to be insensitive (no visible trend) to the TCS presence in the studied concentrations, while the (PEI/PSS)_5_ sensor displays only significant sensitivity in the 10^−5^–10^−7^ M concentration range. Regarding the (PAH/GO)_5_ sensor (Figure 3I), it presents almost constant values of reactance at 25,119 Hz. Analyzing the data plotted individually and only concerning data discrimination (discussed in Section 3.2), the most “efficient” sensor, for DW, seemed to be the (PAH/GO)_5_.

Concerning the TCS detection in MW solutions, the reactance spectra, represented in Figure 2IIa,c, revealed analogous behavior for both gold IDE and (PEI/PSS)_5_ sensors. In the present case (Figure 3II), the loss tangent at fixed frequencies, was used as the transducing variable. The frequency values chosen were 63, 100, and 16 kHz, respectively, for the gold IDE, (PEI/PSS)_5_, and (PAH/GO)_5_ sensors. It should be remarked that all sensors tended to follow a decrease in the loss tangent with decreasing TCS concentrations.

Overall, for the EF, the (PEI/PSS)_5_ sensor was shown to be the most sensitive in the studied TCS concentration range. At frequencies between 1 and 10 kHz, the reactance spectra presented a good response discrimination (Figure 2IIIc). The normalized responses at 3981 Hz as exhibited in Figure 3III, clearly shows an electrical resistance trend with increasing TCS concentrations. It is important to remark, concerning the sensitivity of the sensor, that TCS was being detected/measured in a range of concentrations in accordance with real samples. For instance, the results showed that for the EF matrix, as the TCS concentrations increased, the measured electrical resistance also increased in the range of 10^−8^–10^−12^ M, in accordance with the reported for wastewater samples [10,11,12], supporting its potential field applicability. Should also be referred that the EF matrix has a high amount of anions, Table 1, which directly contributes to the increase of electrical charge that can flow between electrodes, EF conductivity takes a value of 1400 μS/cm [2]. Concerning the gold IDE sensors, the electrical response was operationally difficult to obtain, due to the high conductivity of the medium, and only lower measurable values of resistance were obtained, as can be observed in Figure 3III. The EF matrix is composed of a high concentration of ions, such as Zn and S (Table 1), that interact with the gold electrodes, thus promoting an overload of electrical resistance and contributing to sensor damage during the electrical measurements (optical microscopy images, in Supplementary Material Figure S6).

### 3.2. Principal Component Analysis: Sensor Capabilities

**Figure 4:**
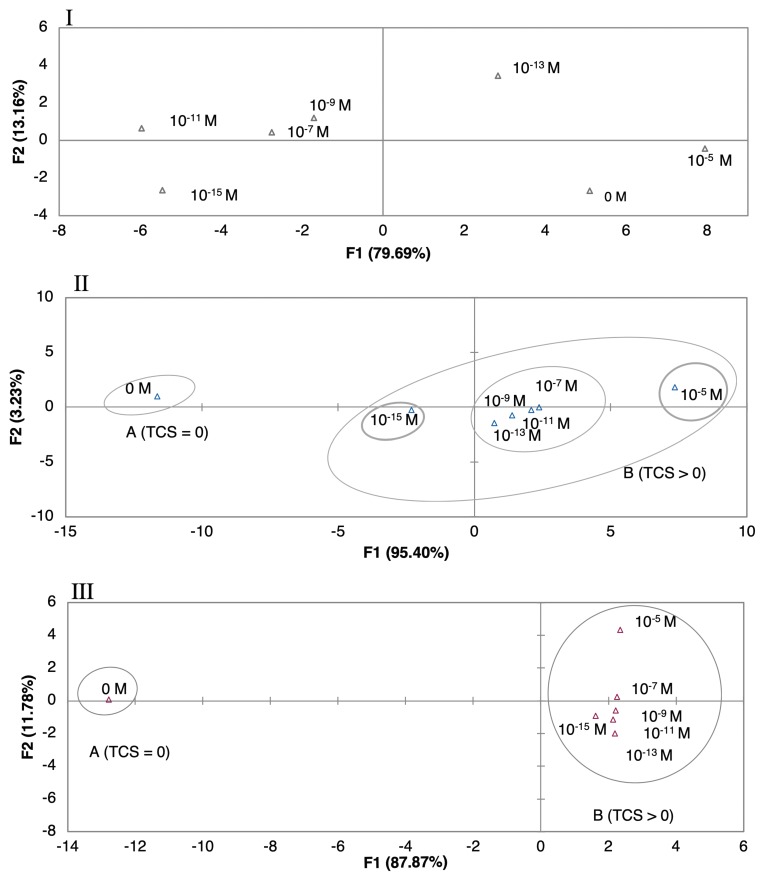
PCA plots of TCS concentrations (10^−5^–10^−15^ M) distinguished by (**I**) the (PAH/GO)_5_ sensor for DW, (**II**) the gold IDE sensor for MW, and (**III**) the (PEI/PSS)_5_ sensor for EF.

In this section three questions will be addressed: 1) Are the tested sensors able to detect concentrations of TCS equal and higher than zero? 2) Are the tested sensors able of distinguish different TCS concentrations? 3) Do the tested sensors produce PCA plots with observable patterns and trends according to TCS concentrations? To look for sensor “discrimination” from different concentrations of TCS, on different matrices, the principal component analysis (PCA), a multivariate analysis technique, was performed after data normalization (Z type). The PCA was used to explore the sensors sensory attributes to produce different patterns for the range of TCS concentrations. The PCA was applied for the most sensitive sensor, regarding impedance normalized data (average of three loops impedance measurements). The PCA with the best score plots obtained for the sensors when immersed in DW, EF, and MW matrices, respectively, are presented in Figure 4I,II,III. The characterization associated to the best sensor is the one where the variations in the plot among TCS concentrations are more significant [48]. The first component F1, explained the greatest data variation and was considered the most important, explaining more than 79.69% of the variance. With the exception of the measurements on DW (Figure 4I), PCA plots produced clearly distinguished the sample matrices without TCS from the samples with TCS (Figure 4II,III).

Regarding the DW experimental matrices (Figure 4I), although the PCA plot distinguished the different concentrations, a trend of TCS concentration per principal components according to concentrations was not achieved. In the case of the (PAH/GO)_5_ sensor, the non-doped solution was in the same quadrant of the higher TCS concentration. A concentration pattern and trend, related to TCS concentrations, was visible for the data plot obtained by the gold IDE sensor (Figure 4II), where eigenvectors points tended to increase over the range of concentrations. The first two principal components, F1 and F2, explained 98.63% of the total variance. Even more, this distinction was better when compared to the one obtained by the thin-films sensors. For the EF matrix, the best discrimination among concentrations was achieved by the (PEI/PSS)_5_ sensor (Figure 4III). It can be observed that TCS concentrations produced a clear trend between 10^−5^ and 10^−13^ M. The first two principal components F1 and F2 accounted for 99.65% of the total variance. This sensor was capable of detection and semi quantification, having a clear trend and pattern within the PCA plot. Considering MW and EF experimental matrices, there was an evident pattern, where the concentration of the non-doped solutions (TCS = 0) might be regarded as an outlier point, being the nearest concentration, in both cases 10^−15^ M. Additionally, for EF the effect of the number of bilayers was tested for the most sensitive sensor obtained, (PEI/PSS)_5_. The PCA plots, showed that five bilayers revealed better response (PCA separation to TCS concentrations) and signal intensity for impedance, when compared to the ones with 10 or 20 bi-layers of (PEI/PSS)_5_ (PCA plots in Supplementary Material Figures S7 and S8). This could be due to the thickness increase with the number of bilayers, which led to the increase of the distance between the gold electrode and the outer layer of film that interacted with the TCS molecules. As the film thickness increased, the capacitance decreased, reducing the electrical signals intensity measured in TCS solutions with different concentrations, i.e., for two distinct concentrations the difference of any measured electric property was smaller. The decrease in the double-layer capacitance was associated with a higher resistance imposed to electron spillover, where this phenomenon reduces the metal capacitance according to the jellium model [49].

**Figure 5:**
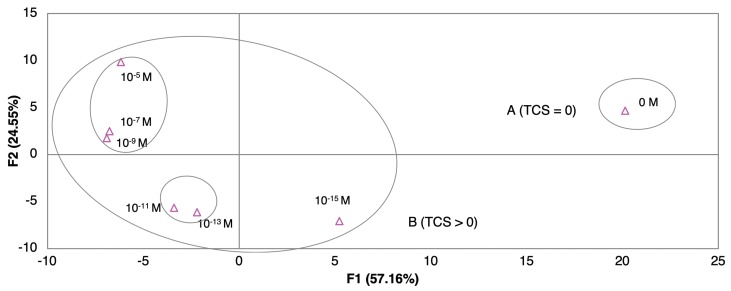
Principal components analysis (PCA) plot of TCS concentrations (10^−5^–10^−15^ M) distinguished with an electronic tongue sensor for EF.

The combination of sensor arrays was expected to improve the capability of TCS concentration discrimination. The array of sensors composed by the sensor devices without any film deposited (gold IDE) and with (PAH/GO)_5_, (PEI/PSS)_5_, (PEI/PSS)_10_, and (PEI/PSS)_20_ thin-films deposited, was used to test the e-tongue concept and to analyze its ability to produce discrimination among TCS concentrations in the EF matrix. The respective PCA plot, Figure 5, clearly distinguished the sample matrices without TCS from the samples with TCS. A concentration pattern and trend, related to TCS concentrations, can be seen on the PCA plot along the main axis. The first two principal components F1 and F2 accounted for 81.71% of the total variance. As it was observed in Figure 4II,III, the data followed the same direction, from region A (TCS) = 0 to region B where (TCS) > 0, proving that the electronic based in those sensor devices was able to distinguish between non-doped wastewater and doped TCS wastewater, producing an observable pattern and trend regarding the different concentration values. 

### 3.3. Sensorial Layers’ Evaluation—Sensor Stability

Despite the positive response behavior of the thin-films sensors in the presence of the TCS molecules in different aqueous matrices, both individually and in an array tell us that we were on the right track for TCS detection, there is a need to evaluate the stability of the thin-films in order to confirm that the positive results are due to the presence of TCS in the aqueous matrix and not to the loss of polyelectrolyte molecules from the films by desorption. It is known that the LbL film stability is related with the electrostatic interactions, in the order of 100 kJ/mol, between the opposite ionized groups of cationic and polyelectrolyte molecules [50,51]. Therefore electrostatic interactions play a vital role in the adsorption of these molecular bilayers but are also strongly pH dependent since the degree of ionization of each polyelectrolyte is a function of pH [50,51]. Having in mind the goal of developing a sensor dedicated to detect a specific molecule in complex media that can present different pH, the electrostatic interactions must be taken into account. Moreover, the salts and/or other elements present in the environmental matrices, change the degree of ionization of LbL films and of molecules involved [52] and therefore affecting the electrical properties (e.g., impedance) of the sensor. 

**Figure 6:**
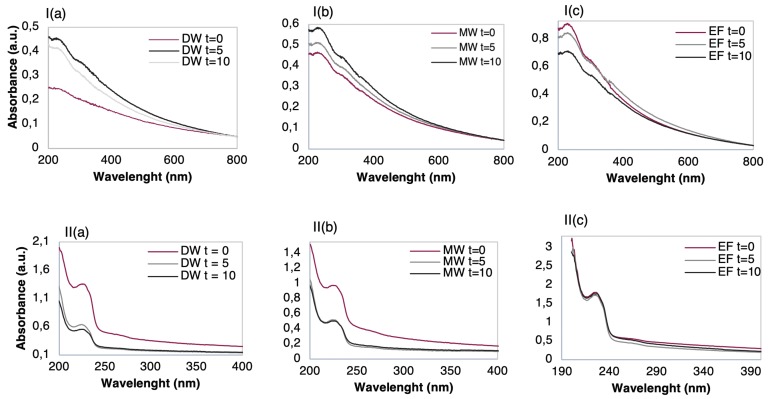
UV-Vis spectra of (**I**) (PAH/GO)_5_ films and (**II**) (PEI/PSS)_5_ before and after immersions times (t = 0, 5, and 10 min) in 10^−9^ M TCS solutions of: (**a**) DW; (**b**) MW, and (**c**) EF.

Additionally, the ionic elements present in the matrices, can be adsorbed onto the polyelectrolyte’s layers, changing the electrical properties of the sensor. Thus, in order to ascertain which sensor holds the best features for the detection of TCS with no loss/desorption of thin-film layers and no irreversible adsorption of TCS onto thin-film, the adsorbed amount on the different thin-films was analyzed before and after the thin-films to be immersed on the different TCS aqueous matrices. The adsorbed amount per unit of area can be easily estimated by measuring the UV-visible spectra of the films since they absorb in this wavelength region and TCS presents two main absorbance bands at 230 and at 280 nm. In Figure 6 the UV-vis absorbance spectra of the LbL films are shown, before and after their immersion (t = 0, 5, and 10 min) into 10^−9^ M TCS aqueous solutions, prepared with DW, MW, and EF (see the Materials and Methods section). These obtained spectra were plotted together by the type of film and aqueous matrix, thus allowing for an insight on the effects of adsorption and/or desorption occurring on each LbL thin-film. Accordantly, if the absorbance is seen to increase compared to the t = 0 is likely that TCS molecules are being adsorbed on the thin-film outermost surface. Contrarily, if the absorbance is seen to decrease when compared to the spectrum measured before immersion of the film on the aqueous matrix, t = 0, it is likely that polyelectrolyte molecules are being desorbed from the thin-film. This desorption is related to losses in the electrostatic interactions with a consequence of changes in the electrical impedance measured during the sensing procedure. By analyzing the spectra of the (PAH/GO)_5_ thin-film displayed in Figure 6Ia–c it is possible to observe that in both DW (a) and MW (b) there was adsorption of TCS molecules on the films being more significant in the case of DW. Additionally, in the DW, results show that a slight desorption took place when the film was immersed from five to ten minutes. This could be due to the more surface-located layers not being completely adsorbed during the film preparation, and when immersed for a second time suffer a washing process, resulting in a loss of previous adsorbed matter [53]. The adsorption phenomenon in these cases holds an additional relevance aside from the detection factor, as it can be used for removing TCS from solutions. 

According to spectra of Figure 6Ia,b, the GO outer layer did not seem to be deeply influenced by pH, given that both waters have different pH values (5.7 for MW and 7.2 for DW, Table 1), and there was adsorption, nonetheless. Moreover, given the chemical structures of the TCS molecule and the polyelectrolyte (GO), the adsorption observed could be a result of either the formation of hydrogen bonds or from the interactions of π–π stacking [39]. In the case of EF, Figure 6Ic, desorption took place since the absorbance decreased as the immersion time in the EF matrix increased. This behavior could be attributed to the abundant presence of ions such as Mg^2+^, Na^+^, and SO_4_^2−^ (Table 1), which strongly interact with the GO outer layer of, facilitating the change of charge within the GO layer, from negatively charged to a predominantly neutral one. By turning neutral, the strong electrostatic interactions between the GO outer layer and the remaining layers were weakened, which in turn leads to polyelectrolyte molecules detachment as shown in Figure 6Ic [54]. The effect of pH on the degree of ionization of PAH and GO has been already discussed in the literature [50,55,56]. In fact, it was shown that although at higher pH GO is electrically charged, the PAH film molecules lost a large amount of charge, leading to both PAH and GO desorption. Due to this fact, this type of film (PAH/GO)_5_ should not be considered as a sensing film for future works containing EF.

From the characterization of UV-vis spectra of (PEI/PSS)_5_ thin-films, Figure 6II, there was a clear effect of film desorption in the cases of DW (a) and MW (b). While for the DW the observed desorption increased with the immersion time, for the MW, although desorption to be significant after five minutes, remained seemingly constant after the second immersion, suggesting that a desorption plateau was reached. As desorption was observed in both DW and MW, (PEI/PSS)_5_ thin-films should not be elected as sensing films for these aqueous matrices.

**Figure 7:**
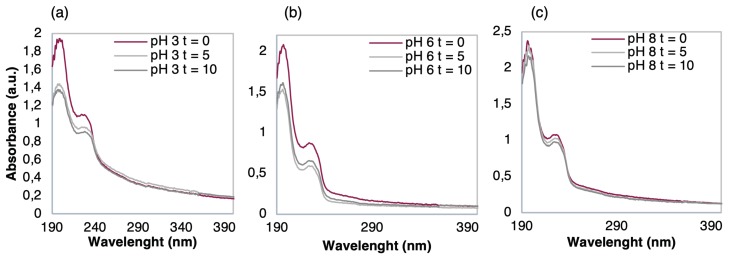
UV-Vis spectra of (PEI/PSS)_5_ films before and after immersions times (t = 0, 5, and 10 min) in 10^−9^ M TCS solutions in EF with a pH of (**a**) pH = 3, (**b**) pH = 6, and (**c**) pH = 8.

Regarding the thin-films in EF, the spectra of Figure 6IIc show that the absorbance between immersion times from t = 0 to t = 10 min slightly varied. This indicates that there was no significant adsorption either desorption, i.e., TCS adsorption or film losses. It is also possible to infer that pH plays a fundamental role in the adsorption and desorption phenomena regarding (PEI/PSS)_5_ LbL films. According to [57], the PEI degree of ionization is strongly pH dependent but the PSS is a strong polyelectrolyte, with a pKa near 1 [52,58], it can be influenced by solution pH due to the presence of a sulfonate group in its chemical structure and furthermore, reaching a more stable state at pH > 7 [59]. Therefore, not only due to this last referred reason but also due to the mineral waters (both have pH < 7), while for EF (pH above 8, see Table 1) the (PEI/PSS)_5_ LbL films were highly stable. To better understand the involved phenomena, a subsequent study about the stability of (PEI/PSS)_5_ thin-films was conducted by measuring the absorbance spectra of (PEI/PSS)_5_ thin-films before and after to be immersed during different periods in EF matrices with different pH, namely, 3, 6, and 8, see Figure 7a–c, respectively. 

Clear desorption occurred after immersion in Figure 7a,b while in Figure 7c a seemingly constant pattern happened among the three spectra. Furthermore, as pH decreased in the TCS solutions from pH 3 to pH 6, the phenomenon of desorption increased, suggesting that pH had a direct effect on the interaction between the thin-film and TCS. The behaviors displayed in Figure 7 further show that the absorbance of TCS onto the outer layer of PSS was deeply influenced by pH, confirming what was observed in Figure 6IIc. At pH 8, the film exhibited a stable behavior, due to PSS being fully charged and PEI maintaining about 40% of its electrical charge, which is sufficient to maintain the polyelectrolyte layers adsorbed in the LbL film and, therefore, no desorption occurred. As the outermost layer of the (PEI/PSS)_5_ thin-film was negative and, at high pH, the TCS was negatively charged, the TCS molecules were repelled by the films and were not adsorbed on its surface. Thus, neither desorption nor adsorption phenomena were observed.

### 3.4. Electronic Tongue—Sensor Sensitivity and Resolution

**Figure 8:**
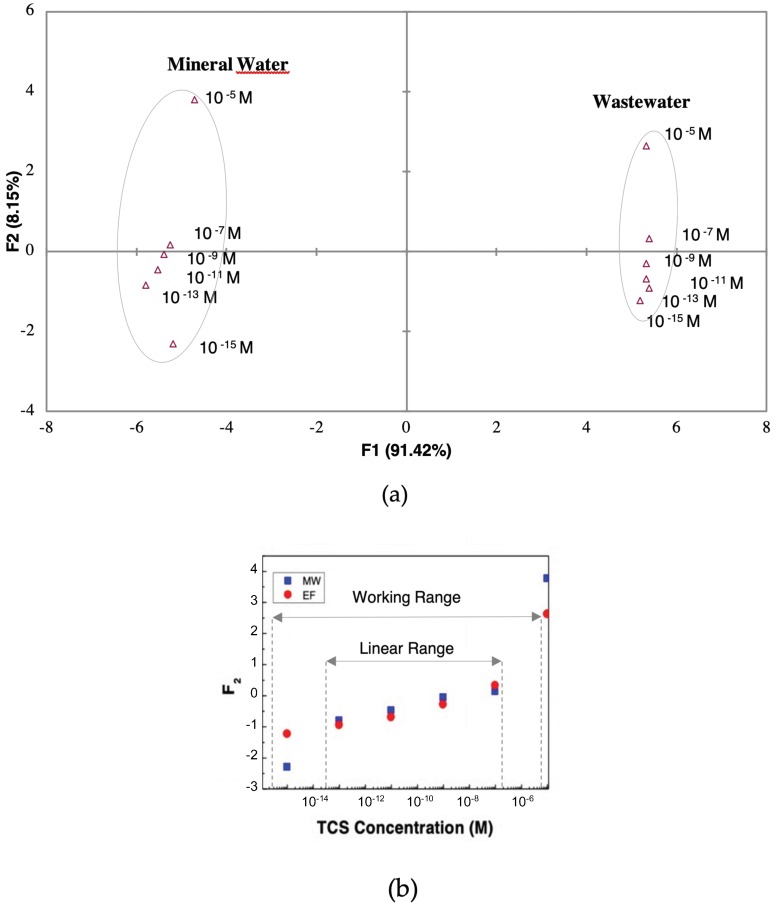
(**a**) PCA plot of TCS concentrations (10^−5^–10^−15^ M) distinguished the gold IDE sensor for MW and the (PEI/PSS)_5_ sensor for EF and (**b**) ((a) PCA data) principal components F2 factor scores as a function of TCS concentrations for the gold IDE sensor in MW and the (PEI/PSS)_5_ sensor in EF.

The results of the previous sections indicate that the (PAH/GO)_5_ LbL sensor should be applied to acid aqueous matrices with lower ionic strengths, while the (PEI/PSS)_5_ LbL sensor should be used with alkaline aqueous matrices with higher ionic strengths. Additionally, the gold IDE sensor should be applied to medium ionic strength in neutral solutions. Thus, to the environmental aqueous matrices under study, an array of sensors, composed by the gold IDE and (PEI/PSS)_5_ sensors was set in order to understand if the e-tongue concept were capable of “tasting”, through the impedance data, MW and EF, doped with TCS (Figure 8).

The PCA plot corresponding to the use of the adequate sensor devices to the matrix is presented in Figure 8a. This plot clearly distinguished the MW from EF matrix. Inside each matrix PCA region it was also possible to discriminate the TCS concentrations across the principal component F_2_. In Figure 8b the values of F_2_ were plotted as a function of TCS concentrations for both types of matrices, and a linear relation and working range of the e-tongue was attained. The principal component F_2_ linear tendency suggests that F_2_ data could be used to determine the sensitivity, the slope of a linear function, of the sensor. Analyzing F_2_ curves, in detail, one could infer a linear range between 10^−13^ and 10^−7^ M of the TCS concentrations. Considering the data within this concentration range, the F_2_ feature plot vs. the logarithm of the concentration, allows fitting the plotted data points to a straight line with a slope, $\frac{\Delta F2}{\Delta \;logC}=0.19\;\pm 0.02$, which corresponds to the sensor sensitivity. Additionally, the sensor resolution (smallest concentration that can be detected) could be found near the smallest concentration (C_s_). The C_s_ value was 0.1 pM while the minimum value that could be measured was 0.02, therefore, $logC=\frac{0.02}{0.19}$ meaning, ΔlogC=logC−logCs and, thus, C − Cs ≈ 0.13 pM, which corresponded to the sensor resolution of 0.13 pM (> 0.3 pg/L). The smallest concentration that could be measured was 10^−13^ M and the range of detection of 10^−13^ to 10^−7^ M.

## 4. Conclusions

An electronic tongue, consisting of an array of sensors based on uncoated IDE and coated with different LbL films was shown to be able to detect and quantify triclosan trace concentrations, within the range of 10^−15^–10^−5^ M, in a MW and in EF matrices, by measuring the impedance spectra of each sensor device. The thin-film stability tests related with adsorption/desorption phenomena revealed that some of the films were not appropriate as TCS monitoring tools as they were highly influenced by both matrices’ ion composition and pH. The experimental data suggests the potential of (PEI/PSS)_5_ LbL films combination to be employed in alkaline aqueous matrices, such as wastewater, with high ionic strength. This is contrary to the gold IDE (uncoated) sensors that reacted with ions in the solutions, were damaged by S and Zn elements, and might be considered only to analyze neutral solutions with low ionic strengths. In fact, the choice of more stable sensing layers is the key to develop quantitative e-tongues and, in the case of LbL films, that stability is only achieved when strong polyelectrolytes are employed in its preparation. 

In this sense, the experimental data, analyzed through a PCA, supported and demonstrated the sensor’s ability and potential to distinguish between aqueous matrices and to discriminate TCS concentrations using the principal component F2. For an experimental/environmental range of triclosan concentrations of 10^−13^–10^−7^ M (0.3–30,000 ng/L), the e-tongue presents a sensitivity value of 0.19 ± 0.02 and a resolution of 0.13 pM. Lastly, observing the main conclusions on the thin-film stability and PCA discrimination through MW and EF, the e-tongue array studied could be used as an “analytical” sensor for the detection and quantification of TCS in the monitoring of environmental aqueous matrices. 

## Figures and Tables

**Table 1:** Characterization of the environmental matrices under study.

	DW	MW	EF *
pH	7.2 ± 0.1	5.7 ± 0.3	8.4 ± 0.1
Conductivity (μS/cm)	46.5 ± 0.7	113 ± 37	1400 ± 500
ICP analysis (mg/L)			
Ca	-	0.75 ± 0.1	39.1 ± 2.8
Cu	-	-	0.02 ± 0.01
K	-	-	22.9 ± 0.4
Mg	-	7.3 ± 0.6	18.2 ± 5.1
Na	-	1.7 ± 0.1	161 ± 58
P	-	-	2.2 ± 0.2
S	-	-	32.9 ± 8.6
Zn	-	-	0.07 ± 0.02
IC analysis (mg/L)			
Cl^−^	-	9.3 ± 0.4	488.7 ± 593.2
SO_4_^2−^	-	1.3 ± 0.3	84.1 ± 92.4

DW—deionized water; MW—mineral water; EF—wastewater; * collected between September and December 2018.

**Table 2:** Characteristics of the thin-films deposited on BK7 solid support with gold interdigitate electrodes to characterize the aqueous solutions matrices.

Aqueous Matrices	Thin-Film Combinations	Number of Bilayers
DW	PAH/GO and PEI/PSS	5
MW	PAH/GO and PEI/PSS	5
EF	PAH/GO	5
	PEI/PSS	5, 10, and 20

## Supplementary Materials

The following are available online at https://www.mdpi.com/2079-4991/10/4/640/s1, Table S1: Triclosan physical and chemical characteristics; Table S2: Concentration guidance: molar, µg/, ng/L, and pg/L; Figure S3.1: Sensor device used: glass BK7 solid support with deposited gold interdigitated electrodes from DropSens (Llanera Asturias, Spain); Figure S3.2: Scheme of the sensor’s preparation for the electronic tongue array; Figure S4: Reactance (imaginary) impedance spectra of sensor device of the gold IDE sensor, (PAH/GO)_5_ sensor, and (PEI/PSS)_5_ sensor, immersed in deionized water, mineral water and wastewater with 10^−15^ M of TCS (related to the measurements in Figure 2); Table S5.1: Impedance data measurements, reactance (imaginary), used for the normalization of Plots I in Figure 3; Table S.5.2: Impedance data measurements, loss tangent, used for the normalization of Plots II in Figure 3; Table S.5.3: Impedance data measurements, resistance, used for the normalization of Plots III in Figure 3; Figure S6: Optical microscopy images of gold interdigitated electrodes without coating, gold IDE; Magnitude: (a) 4×, (b)10×, and (c) 40×, analyzed in wastewater; Figure S7: PCA plot of TCS concentrations (10^−5^–10^−15^ M) distinguished with (PEI/PSS)_10_ sensor for wastewater; Figure S8: PCA plot of TCS concentrations (10^−5^–10^−15^ M) distinguished with (PEI/PSS)_20_ sensor for wastewater
