# Supplementary material for: Polyelectrolyte Based Sensors as Key to Achieve Quantitative Electronic Tongues: Detection of Triclosan on Aqueous Environmental Matrices
Source: Nanomaterials (Basel). 2020 Mar 29;10(4):640. doi: 10.3390/nano10040640 (PMC7221897; doi:10.3390/nano10040640)
Supplement: Supplementary file 1 [file nanomaterials-10-00640-s001.pdf]

Supplementary Material

Polyelectrolyte Based Sensors as Key to Achieve Quantitative Electronic Tongues: Detection of Triclosan on Aqueous Environmental Matrices

Table S1. Triclosan physical and chemical characteristics.

Chemical structure	Formula	Molar mass g mol $^{-1}$	Log K_{ow}	pKa	Solubility in water mg L^{-1}	Polarizability Å ^{3}	CAS-No
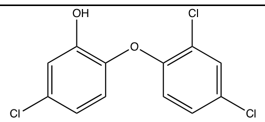<br>5-chloro-2-(2,4-dichlorophenoxy)phenol	$C_{12}H_7Cl_3O_2$ *	289.54 *	4.76	7.9	10 (20°C)	26.96	3380-34-5 *

References: http://pubchem.ncbi.nlm.nih.gov/; * https://chemicalize.com/.

Table S2. Concentration guidance: molar, μg/, ng/L and pg/L.

M	*μg/L*	*ng/L*	*pg/L*
10^{-5}	3000		
10^{-6}	300		
10^{-7}	30		
10^{-8}	3		
10^{-9}	0.3		
10^{-10}		30	
10^{-11}		3	
10^{-12}		0.3	300
10^{-13}			30
10^{-14}			3
10^{-15}			0.3

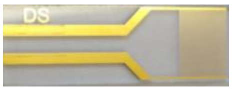

Figure S3.1. Sensor device used: glass BK7 solid support with deposited gold interdigitated electrodes from DropSens (Llanera Asturias, Spain).

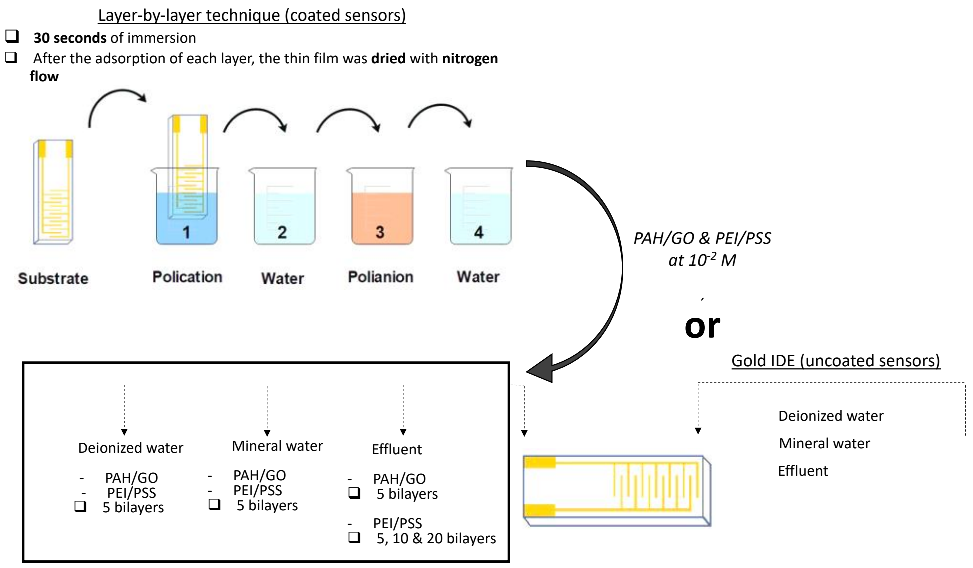

Figure S3.2 Scheme of the sensor's preparation for the electronic tongue array.

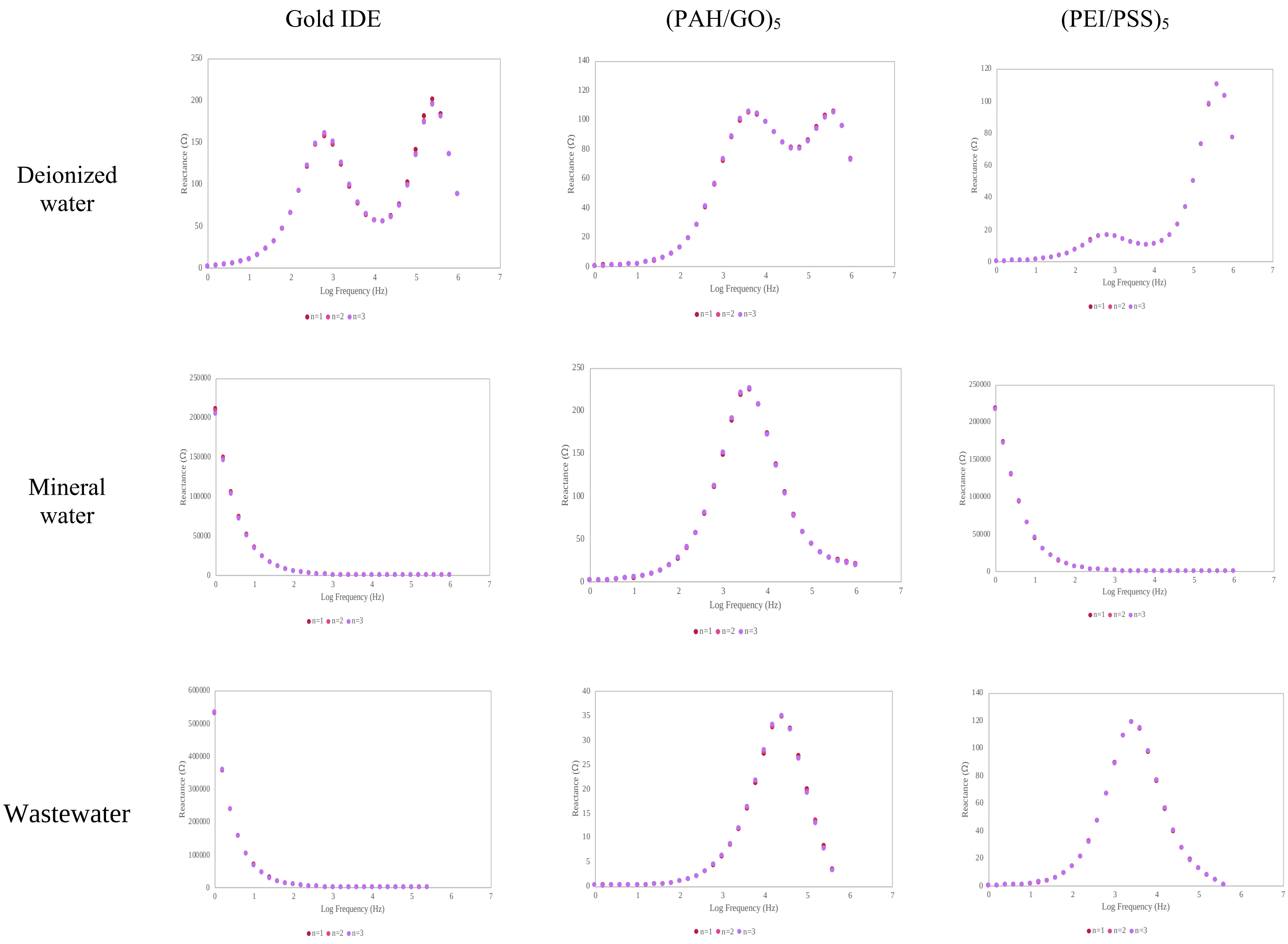

Figure S4. Reactance (imaginary) impedance spectra of sensor device of gold IDE sensor, $(PAH/GO)_5$ sensor and of $(PEI/PSS)_5$ sensor, immersed in deionized water, mineral water and wastewater with 10^{-15} M of TCS (related to the measurements in Figure 2).

Table S5.1 Impedance data measurements, reactance (imaginary), used for the normalization of Plots I in Figure 3.

		Fixed Frequency (Hz)					
		6.3		25119		25119	
	TCS (M)	Gold IDE		$(PAH/GO)_5$		$(PEI/PSS)_5$	
		Average	*SD*	*Average*	*SD*	*Average*	*SD*
Reactance (imaginary) (Ω) for deionized water	0	13.035	0.654	87.201	0.112	16.837	0.021
	10^{-15}	7.466	0.041	84.483	0.030	16.338	0.004
	10^{-14}	7.508	0.028	84.460	0.048	16.576	0.004
	10^{-13}	7.270	0.032	83.752	0.092	16.616	0.005
	10^{-12}	7.085	0.027	83.488	0.053	16.379	0.012
	10^{-11}	7.035	0.030	83.668	0.017	16.461	0.003
	10^{-10}	7.051	0.041	83.694	0.029	16.508	0.008
	10^{-9}	6.976	0.019	83.889	0.017	16.541	0.003
	10^{-8}	7.015	0.029	83.834	0.023	16.638	0.002
	10^{-7}	6.939	0.023	84.166	0.015	16.538	0.011
	10^{-6}	6.939	0.014	85.315	0.136	18.439	0.100
	10^{-5}	6.795	0.020	87.068	0.086	21.371	0.044

SD – standard deviation.

Table S5.2. Impedance data measurements, loss tangent, used for the normalization of Plots II in Figure 3.

		Fixed Frequency (Hz)					
		63095		16000		100000	
	TCS (M)	Gold IDE		$(PAH/GO)_5$		$(PEI/PSS)_5$	
		Average	SD	Average	SD	Average	SD
Loss Tangent (a.u) for mineral water	0	6.586	0.038	2.956	0.000	4.454	0.001
	10^{-15}	6.613	0.009	2.961	0.005	4.600	0.008
	10^{-14}	6.547	0.009	2.892	0.003	4.578	0.006
	10^{-13}	6.492	0.010	2.877	0.003	4.517	0.002
	10^{-12}	6.413	0.005	2.876	0.005	4.522	0.002
	10^{-11}	6.382	0.001	2.877	0.006	4.423	0.003
	10^{-10}	6.347	0.001	2.863	0.003	4.457	0.001
	10^{-9}	6.327	0.002	2.887	0.002	4.419	0.001
	10^{-8}	6.256	0.001	2.870	0.003	4.386	0.004
	10^{-7}	6.272	0.003	2.863	0.004	4.388	0.002
	10^{-6}	6.210	0.003	2.854	0.004	4.329	0.004
	10^{-5}	5.942	0.036	2.826	0.002	4.206	0.009

SD – standard deviation.

Table S5.3. Impedance data measurements, resistance, used for the normalization of Plots III in Figure 3.

		**Fixed Frequency (Hz)**					
		**100000**		**16000**		**3981**	
	TCS (M)	Gold IDE		$(PAH/GO)_5$		$(PEI/PSS)_5$	
		Average	SD	Average	SD	Average	SD
**Resistance (Ω) for wastewater**	0	40.920	0.057	98.217	0.338	149.621	1.055
	10^{-15}	40.781	0.041	94.688	0.561	150.891	0.286
	10^{-14}	41.064	0.027	94.458	0.289	151.813	0.168
	10^{-13}	41.241	0.066	93.091	0.145	153.332	0.217
	10^{-12}	40.712	0.011	93.272	0.095	154.904	0.241

10^{-11}	40.906	0.032	93.219	0.076	154.231	0.124
10^{-10}	40.716	0.029	93.034	0.068	155.732	0.124
10^{-9}	40.596	0.030	92.116	0.047	156.077	0.153
10^{-8}	40.645	0.043	93.234	0.066	158.578	0.192
10^{-7}	40.794	0.023	92.224	0.039	158.783	0.142
10^{-6}	40.718	0.009	93.262	0.004	161.490	0.401
10^{-5}	41.151	0.021	92.677	0.218	167.916	0.771

SD – standard deviation.

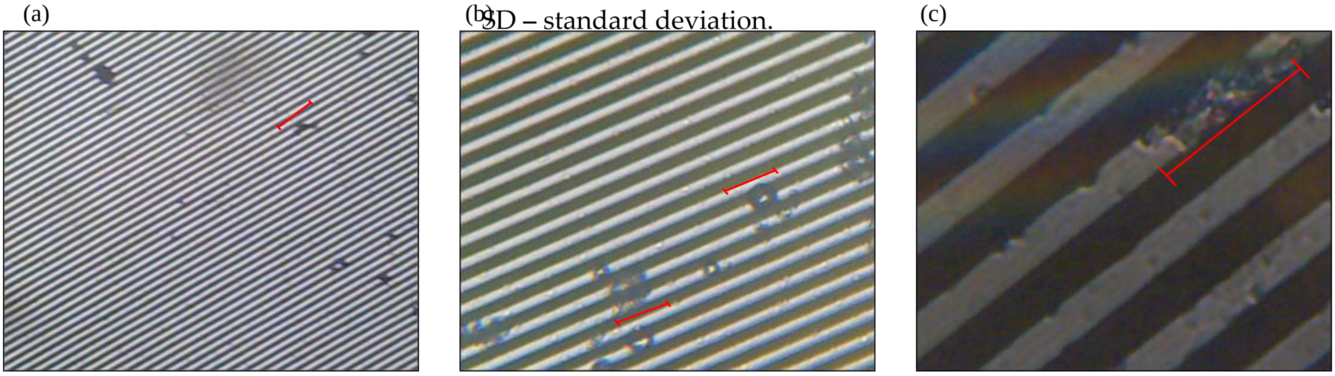

Figure S6. Optical microscopy images of gold interdigitated electrodes without coating, gold IDE; Magnitude: (a) 4×, (b)10× and (c) 40×, analysed in wastewater.

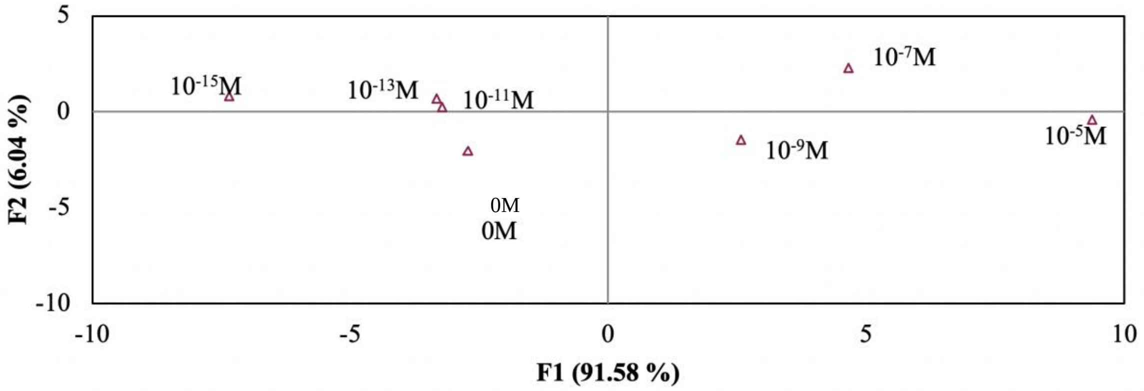

Figure S7. PCA plot of TCS concentrations (10^{-5}-10^{-15}M) distinguished with (PEI/PSS)$_{10}$ sensor for wastewater.

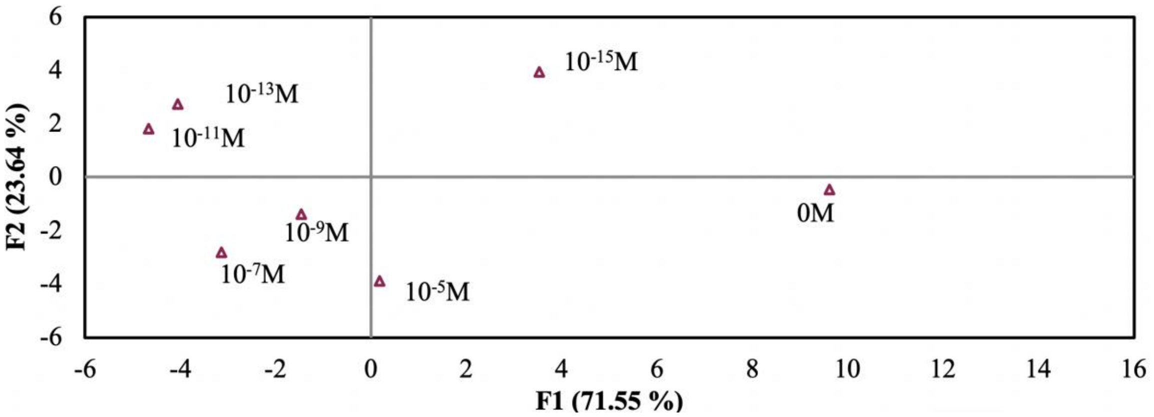

Figure S8. PCA plot of TCS concentrations (10^{-5}-10^{-15}M) distinguished with (PEI/PSS)$_{20}$ sensor for wastewater.
